# *Cestrum strigilatum* (Ruiz & Pavón, 1799) B chromosome shares repetitive DNA sequences with A chromosomes of different *Cestrum* (Linnaeus, 1753) species

**DOI:** 10.3897/CompCytogen.v11i3.13418

**Published:** 2017-08-03

**Authors:** André Luís Laforga Vanzela, Adriano Alves de Paula, Carolina Cristina Quintas, Thiago Fernandes, Joana Neres da Cruz Baldissera, Thaíssa Boldieri de Souza

**Affiliations:** 1 Laboratory of Cytogenetics and Plant Diversity, Center for Biological Sciences, State University of Londrina, Londrina, 86051-980, Paraná, Brazil

**Keywords:** FISH, karyotypes, microdissection, rDNA, Solanaceae, supernumerary chromosomes

## Abstract

Species of *Cestrum* (Linnaeus, 1753) have shown large diversity in the accumulation and distribution of repetitive DNA families, and B chromosomes have been described in seven species. Some types of repetitive DNA were identified in A and B chromosomes in species of this plant group, such as AT-rich SSR, 35S and 5S rDNA, C-Giemsa and C-CMA/DAPI bands and retrotransposons. To increase our understanding of the relationships of A and B chromosomes, the B of *C. strigilatum* Ruiz & Pavón, 1799 was microdissected, amplified and hybridized *in situ* against chromosomes of this species, and in six other species of this genus. FISH signals were observed in whole the B of *C. strigilatum*, including stretches of A chromosomes, as well as in some A chromosomes of all tested species. A strong FISH signal was seen adjacent to the 5S rDNA in the proximal region of pair 8 of all species and, due to this, we have searched for 5S rDNA fragments in the microdissected B chromosome. PCR and sequencing data evidenced 5S rDNA deletion along evolutionary pathways of the B of *C. strigilatum*. Although A and B chromosomes displayed redundancy in the repetitive DNA families in different species, the B of *C. strigilatum* seemed to differ from those Bs of other *Cestrum* species by the loss of rDNA fractions. A possible origin of Bs in *Cestrum* was discussed.

## Introduction

B chromosomes have been described as additional and dispensable components of genomes, especially because they show few or no essential genes, abundance of repetitive DNA families, and independent meiotic behavior, without pairing with A chromosomes (Puertas 2002, Jones and Houben 2003). Although new information from the genomic sequencing has improved the knowledge on the B chromosomes of plants (Houben et al. 2014), the exact origin of these chromosomes is difficult to know, and it can be different for each genomic history. It is widely accepted that Bs arise from portions of the A complement by different pathways (Camacho et al. 2000, Jones and Houben 2003), such as chromosome rearrangements and sequence amplifications (Teruel et al. 2010, Valente et al. 2014), or after interspecific hybridizations (Navabi et al. 2011). Since B chromosomes do not exhibit Mendelian inheritance pattern, it is possible that they are maintained in populations by drive mechanisms. Such events are favored by mitotic and/or meiotic instabilities, which can provide an accumulation of Bs in different somatic and germ tissues (Burt and Trivers 2006, Jones 2012).

Approximately 24,000 species of angiosperms (4% of them) have Bs (Levin et al. 2005), and apparently, large genomes could favor or influence the occurrence of B chromosomes (Trivers et al. 2004). This appears to be true for some Solanaceae, since in the genus *Cestrum*, for example, species have 16 chromosomes with up to 12 µm and 20 pg of DNA per diploid set (Paula et al. 2015), and show the highest number of taxa with Bs. These chromosomes have been reported in *C. diurnum* Linnaeus, 1753 (Sobti et al. 1979), *C. parqui* L’Héritier, 1788 × *C. aurantiacum* Lindley, 1844 (Sýkorová et al. 2003), *C. intermedium* Sendtner, 1846, *C. strigilatum* (Fregonezi et al. 2004), *C. parqui*, *C. euanthes* Schlechtendal, 1832 and *C. nocturnum* Linnaeus, 1753, with 1-2, 1-3 and 1-10 B chromosomes, respectively (Urdampilleta et al. 2015). B chromosomes have also been reported for *Nierembergia aristata* David Don, 1833 (Solanaceae), which also has large chromosomes (Acosta and Moscone 2011).

Species of *Cestrum* exhibit a large variation in the occurrence and distribution of repetitive DNA families (Paula et al. 2015), and some of them have already been identified and associated with B chromosomes (Fregonezi et al. 2004). In the hybrid *C. parqui* × *C. aurantiacum*, for instance, the B chromosome contains 35S and 5S rDNA and SSR AT-rich motifs (Sýkorová et al. 2003). Sequences of rDNA were also identified in Bs of *C. parqui*, *C. euanthes* and *C. nocturnum* (Urdampilleta et al. 2015). In *C. intermedium* and *C. strigilatum*, besides C-Giemsa^+^/CMA^+^/DAPI^+^ bands (Fregonezi et al. 2004), the Bs also display hybridization signals with the *Gypsy*-like retrotransposons probe but not with rDNA probes (Fregonezi et al. 2007). The accumulation of data on the Bs of *Cestrum* (Sýkorová et al. 2003, Fregonezi et al. 2004, 2007, Fernandes et al. 2008 and Urdampilleta et al. 2015) have constantly prompted us to extend studies on the composition and behavior of these chromosomes, since this genus seems to be the main model for studies of Bs in the scope of the Solanaceae. The aim of this study was to understand the relationships between the B and A chromosomes of *C. strigilatum*, as well as with A chromosomes of other *Cestrum* species. Additionally, we have also searched any indication on presence of rDNA sequences in this B, in view of the existence of rDNA in Bs of other species of this genus. The discussion addresses aspects about the origin and differentiation of Bs in *Cestrum*.

## Material and methods

Samples of *Cestrum strigilatum*, *C. bracteatum* Link & Otto, 1828, *C. corymbosum* Schlechtendal, 1832, *C. laevigatum* Schlechtendal, 1832, *C. mariquitense* Kunth, 1818, *C. sendtnerianum* Martius, 1846, and *C. schlechtendalii* George Don, 1838 from Brazil are maintained in the greenhouse of the Laboratory of Cytogenetics and Plant Diversity, State University of Londrina, and vouchers are kept at the FUEL herbarium.

Root tips were pre-treated with 0.05% colchicine at room temperature for 4 h, fixed in a solution of ethanol/acetic acid (3:1,v:v) for up to 12 h and stored at -20°C. For conventional staining, samples were softened in 2% cellulase plus 20% pectinase (w:v) at 37°C, hydrolyzed in 1 M HCl for 10 min at 60°C, dissected in a drop of 45% acetic acid, and then squashed. Meiotic cells of *C. strigilatum* samples containing a B chromosome were obtained from young anthers, which were collected, directly fixed and dissected as described above. For both cases, after coverslips removal using freezing in liquid nitrogen, samples were stained in 2% Giemsa for conventional analysis and mounted with Entellan (Merck), or stored in 70% ethanol without staining when samples were for microdissection.

B chromosomes were isolated using an inverted Olympus IX71 microscope, equipped with Narishige micromanipulator. The B chromosome of *C. strigilatum* is three times smaller than those chromosomes of A set, being easily recognized and differentiated. We have dissected only those Bs that were far enough from the As, taking care to avoid contaminants. Five microdissections were made, and in each, 15 chromosomes were transferred to sterile tubes. Samples were treated with 1 µg/mL proteinase K at 60°C for 1 h and 30 min and the product was puriﬁed using phenol:chloroform (1:1, v:v), and quantified in a Nanodrop 2000 (Thermo). Afterwards, DNA of Bs was amplified using a Random Priming® kit (Invitrogen) with biotin-14-dATP for B probe production.

Sequences of 5S rDNA were amplified by PCR using as template the genomic DNA of *C. strigilatum*, and also a pool of 15 microdissected and purified B chromosomes of the same species. To test the reliability of reactions, three PCR repetitions were done using three different samples of microdissected B chromosomes. For this, two set of primers were used to amplify different fragments: 5S-plant-F 5’CACCGGATCCCATCAGAAACT and 5S-plant-R 5’TTAGTGCTGGTATGATCGCA, for NTS region, and UP46-F 5’GTGCGATCATACCAGCRYTAATGCACCGG and UP47-R 5’ GAGGTGCAACACGAGGACTTCCCAGGAGG, for gene coding. PCR were done using a mix containing 2 mM MgCl_2_, 0.4 μM of each primer, 0.2 mM dNTP, 0.2 mM DNA template, 2 U of *Taq* polymerase and ultrapure water to complete 25 μL. To probe labeling, 0.2 mM dNTP was changed by solution containing dGTP (25%), dCTP (25%), dTTP (25%), dATP (17.5%) and bio- or dig-dATP (7.5%). When UP46 and UP47 primers were used, thermal cycler was adjusting to the following conditions: 5 min at 94°C, followed by 35 cycles of 1 min at 94°C, 30 sec at 60°C (genome the template) or 30 sec at 50°C (B chromosome) and 1 min at 72°C, and one end of step 5 min at 72°C. When 5S-plant primers were used the conditions were: 5 min at 94°C, followed by 35 cycles of 1 min at 94°C, 40 sec at 50°C (genome and B chromosome as template) and 1 min at 72°C, and one end of step 5 min at 72°C. To check the presence of 5S rDNA fragments in the B chromosomes, both PCR products were used for FISH. The PCR products were used in a second reaction to produce templates for a Sanger sequencing, using the 3500xL Automatic Sequencer (Applied Biosystems), according to the manufacturer’s procedures. For the sequencing three distinct reactions for each primer (F and R) were done, and repeated once. The consensus sequences were obtained after alignment of 12 sequences, in which quality were tested with Phred/Phrap/Consed software, and consensus sequences were contrasted against GenBank (http://www.ncbi.nlm.nih.gov/blast) to check similarities with other 5S rDNA sequences. For FISH, a mixture of 30 µL containing 100% formamide (15 µL), 50% polyethylene glycol (6 µL), 20× SSC (3 µL), 100 ng calf thymus DNA (1 µL), 10% SDS (1 µL) and 100 ng probes (4 µL), was treated at 70 °C for 10 min, placed on ice and immediately applied to the samples. B and 35S rDNA probes were labeled with biotin by random primers and nick translation, respectively. 5S rDNA probes were labeled with biotin or digoxigenin by PCR. Denaturation/hybridization was performed at 95 °C, 50 °C and 38 °C, ten minutes each, followed by 37 °C overnight in a humidified chamber. Post-hybridization washes were carried out in 6× SSC and 4× SSC/0.2% Tween 20 (> 60% stringency), and the probes were detected using avidin-FITC (green) and anti-dig-rhodamine (red) conjugated. Post detection washes were carried out in 4× SSC/0.2% Tween 20 at room temperature. Slides were mounted in 23 µL antifade solution (90% glycerol, 2.3% DABCO, 2% 20 mM Tris–HCl, pH 8.0, plus 1 µL of 2 μg/mL DAPI and 1 µL of 2.5 mM MgCl_2_).

We have analyzed at least five preparations for each species and of these, at least five metaphases. All the chromosome images were acquired in gray-scale mode using a Leica DM 4500 B microscope equipped with a DFC 300FX camera and overlapped with blue for DAPI, greenish-yellow for FITC and red for rhodamine, using Leica IM50 4.0 software. The images were optimized for contrast and brightness using the GIMP 2.8 Image Editor. To ensure hybridization of the maximum possible sequences in B and A chromosomes, we did not use pre-hybridization or blocking DNA containing excess unlabeled repetitive DNA fractions, which is generally indicated for chromosome painting procedures. This allowed us to ascertain the participation of some A complement DNA fractions in the B composition. Additionally, this allowed us to determine the presence or absence of repetitive sequences in the genomes of other closely related species.

## Results and discussion

### B of *Cestrum strigilatum*

**Figure 1.**
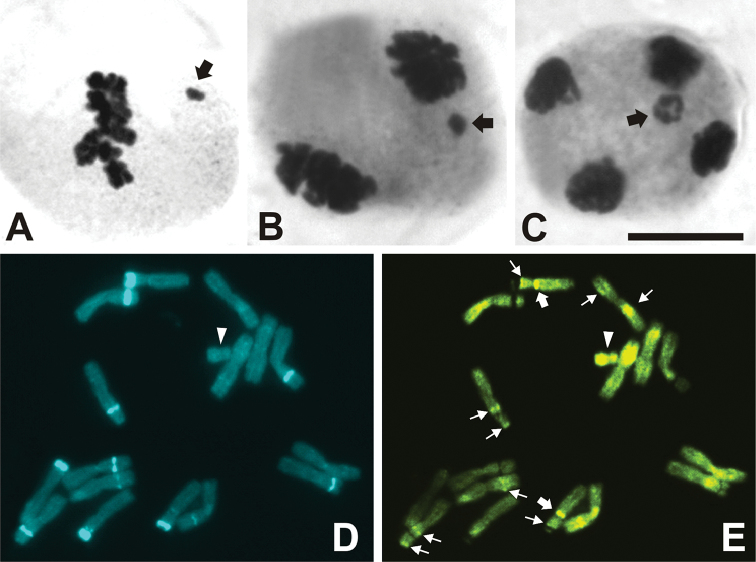
Details of B chromosome of *Cestrum strigilatum* at meiosis, and FISH using the B probe against the karyotype. **A** Metaphase I showing the B as univalent, laterally located (arrow) **B** Anaphase I showing the lagging B univalent moved to one of the complements (arrow) **C** Late anaphase II close to one of the four complements. Note that the chromatids are partially separated (arrow) **D** Mitotic metaphase stained with DAPI. Arrowhead indicates the B **E** Mitotic metaphase hybridized with B probe. Note that some DAPI bands in D appear negative in E, but others do not. Large arrows indicate FISH signal colocalized with 5S rDNA (see also Fig. 2), small arrows point out intercalary and terminal FISH signals, and arrowhead indicates the B completely stained by probe. Bar = 10 µm.

The screening for B chromosomes in the meiosis of *Cestrum strigilatum* showed that B always appears as univalent, without any kind of pairing with A chromosomes (Fig. 1a). In general, Bs of *C. strigilatum* appear displaced from the other chromosomes at metaphase I/anaphase I (Fig. 1a–b, see also Fregonezi et al. 2004), and more centrally located in the second stage of meiosis (Fig. 1c). The small size of the Bs (about three times less than A chromosomes), as well as the displacement from the A chromosomes, provided preparations of sufficient quality to isolate Bs using the microdissection technique, without any contamination with stretches of other chromosomes.

**Figure 2.**
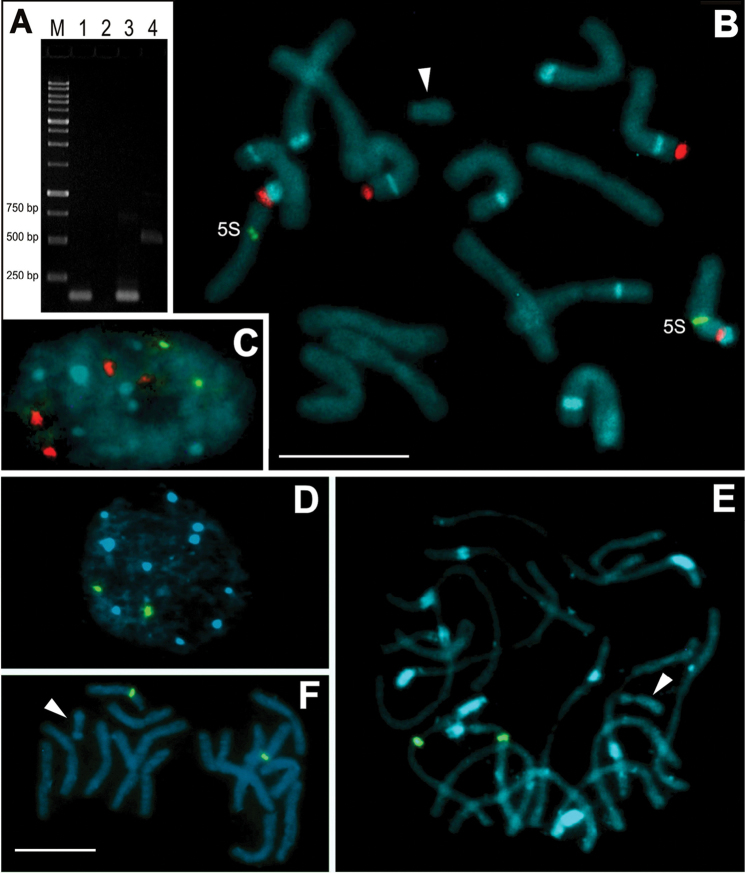
Detection of 5S rDNA by PCR and FISH using probes produced with genomic and B chromosome DNA templates. **A** Electrophoresis gel containing: M = ladder with 250, 500, 750, 1000 bp. Lanes 1 and 3 represent fragments of about 120 bp using the UP primers using the microdissected B chromosome as template (1) and the genome as template (3). Lanes 2 and 4 represent the PCR using 5S-plant primers with microdissected B chromosome as template (see the absence of fragment in lane 2) and with genome as template (see a fragment with about 500 bp length in lane 4 **B–C** Double FISH showing four hybridization sites for 35S rDNA using the p*Ta*71 probe (red) and two sites for 5S rDNA using the UP primer probe of the genome (green) in the chromosomes (**B**) and nucleus (**C**) of *C. strigilatum* **D–F** FISH using the 5S rDNA fragment amplified, using the UP primers and the microdissected B DNA as template. Note only two signals in the nucleus and prometaphase chromosomes of *C. strigilatum* (**D, E** respectively). Note also the absence of hybridization signals in the Bs (arrowheads). Bands in blue color represent AT-rich regions identified by DAPI staining. Bar = 10 µm.

Chromosome painting using the microdissected B as probe was successfully employed in the *Cestrum* species. This procedure was previously used for chromosome painting in species of *Secale* Linnaeus, 1753, *Allium* Linnaeus, 1753 and *Brachyscome* Cassini, 1816 (see Houben et al. 2001), but when we compare data from literature with our case, the absence of blocking with repetitive fractions in FISH made it possible to see many signals on A chromosomes of *C. strigilatum*. The B chromosome of *C. strigilatum* was totally hybridized, and almost all A chromosomes of this species showed hybridization signals in different positions, such as: i) signals adjacent to C-DAPI bands, ii) lightly dispersed signals throughout the chromosomes, and iii) an intense hybridization signals associated with 5S rDNA sites, in the proximal region of long arm of pair 8 (Fig. 1d-e and 2b). Of all the signals produced by the B chromosome-specific probe, the strong hybridization signal in the 5S rDNA region (Fig. 1e) drew our attention, because previous reports indicate the absence of 5S rDNA in the Bs of *C. strigilatum* and *C. intermedium* (Fregonezi et al. 2004). Some repetitive sequences have been described for Bs of different species of *Cestrum*, such as C-Giemsa^+^/CMA^+^/DAPI^+^ bands, rDNA, SSR and *Gypsy*-like retrotransposons, and all of them also appeared in parts of A chromosomes (Sýkorová et al. 2003, Fregonezi et al. 2004, 2007, Urdampilleta et al. 2015). According to Houben et al. (2014), B chromosomes can be more common in species or groups of species whose genomes are involved in intense chromosomal rearrangements. Moreover, large genomes, as well as those involved with chromosome rearrangements and DNA sequence amplifications could favor the arising of B chromosomes (Trivers et al. 2004, Teruel et al. 2010, Valente et al. 2014). This seems to be the case of *Cestrum* species, since they exhibit large genomes and karyotypes with wide diversity in the distribution of repetitive DNA fraction (Paula et al. 2015).

### Detecting 5S rDNA fragments on B chromosome

Previous studies using FISH technique have shown that B of *C. strigilatum* carry no rDNA sequences (Fregonezi et al. 2004). On the other hand, in the B chromosomes of *C. parqui* × *C. aurantiacum* (Sýkorová et al. 2003) and in *C. parqui*, *C. euanthes* and *C. nocturnum* (Urdampilleta et al. 2015), 35S and 5S rDNA signals were detected. To test the colocalization of B probe signals with rDNA clusters, a double FISH using p*Ta*71 and p*Ta*794 clones (containing 35S and 5S rDNA of *Triticum* Linnaeus, 1753, respectively) was done after FISH with the B probe in chromosomes of *C. bracteatum*, *C. corymbosum*, *C. laevigatum*, *C. mariquitense*, *C. sendtnerianum*, *C. schlechtendalii* and also with *C. strigilatum* with one B. Results showed that there was no association between hybridization signals produced by the B and 35S rDNA probes in any species analyzed. Although the B probe signal appeared colocalized with the signal of 5S rDNA in *C. strigilatum* and in the other six species (Fig. 4a–l), the p*Ta*794 probe containing the 5S rDNA fraction did not hybridize with the B chromosome of *C. strigilatum* (Fig. 2b, f, e). The presence of ribosomal sites on B chromosomes is not uncommon, having been reported for Bs of *Crepis capillaris* (Linnaeus, 1753) Wallroth, 1840, Asteraceae (Maluszynska and Schweizer 1989), *Nierembergia aristata*, Solanaceae (Acosta and Moscone 2011) and *Plantago lagopus* Linnaeus, 1753, Plantaginaceae (Dhar et al. 2002). This latter species is remarkable because a large amplification of 35S and 5S rDNA sequences contributed to the origin of B chromosome. In addition, the B chromosome of *P. lagopus* exhibits one satDNA (named PLsatB), which represents approximately 3.3% of the genome of individuals with B, that was originated from amplification of 5S rDNA sequences (Kumke et al. 2016). At least for *Cestrum*, the occurrence of rDNA sequences in the Bs seems to be a plesiomorphic feature, and accordingly, the absence of 35S and 5S rDNA signals in the B of *C. strigilatum* raised an issue: Were rDNA sequences lost during B chromosome differentiation in these two species or do these Bs have an independent origin, without any contribution of rDNA sequences?

**Figure 3.**
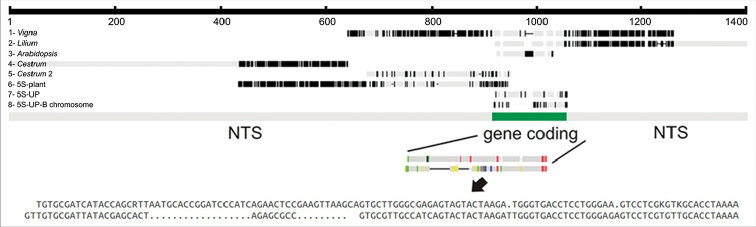
Diagram representing the partial alignment of 5S rDNA sequences of *Vigna* (AP017185.1), *Lilium* (KM117262.1), *Arabidopsis* (AY130622.1), *Cestrum aurantiacum* × *C. parqui* (AY135508.1), *Cestrum psittacinum* (AF495752.1), 5SplantCestrum, 5SUPCestrum and 5SUP-BChrom, for location of gene coding and NTS regions. Note the two internal deletions in the 5S rDNA gene coding of B chromosome sequence (arrow).

PCR was conducted using DNA templates of the genome and microdissected B chromosome of *C. strigilatum*, and two distinct primer sets for amplification of different internal 5S rDNA sequences were used (Fig. 2a). The UP46-F and UP47-R primers were used to amplify the coding region, and the 5S-Plant-R and 5S-Plant-F primers were used for amplification of the less-conserved NTS fragment, including a small stretch of the coding region (Fig. 3). The PCR using the UP primers and the *C. strigilatum* genome as template amplified a fragment with 122 bp length, but when the B chromosome was used as template, the PCR product was a 97 bp fragment (Fig. 2a). For the PCR using the 5S-Plant primers, only the genome template produced amplification, with a fragment containing 513 bp (Fig. 2a). Results of Sanger sequencing have shown three scenarios (Table 1): i) that fragment of 122 bp length of UP primers+genome exhibited high identity with a conserved 5S gene coding of *Vigna angularis* (Willdenow) Ohwi & Ohashi, 1969 and *Lilium tsingtauense* Gilg, 1904, ii) in the second, using UP primers+B template, the fragment of 97 bp length showed a partial coverage with the conserved 5S gene coding of *V. angularis* and *L. tsingtauense*. When these two fragments were alingned, they exhibited 86% identity, but the B chromosome fragment showed two internal deletions (18 and 11 bp in length) separated by a short AGA(A/G)C motif, which seems to indicate a degeneration in the middle of the sequence (Figs 3 and S1). In the third case (5S-Plant primers+genome template), the fragment of 513 bp presented high identity with 5S non transcribed sequences (NTS) of the hybrid *C. aurantiacum* × *C. parqui* and *Cestrum psittacinum* (Stapf, 1828) (Fig. 3 and Suppl. material 1). PCR using 5S-Plant primers using B chromosome as template produced no bands. When these three fragments were biotin-labeled and used in FISH reactions, only hybridization signals in the proximal region of the large arm of pair 8 were detected, i.e., the B chromosome of *C. strigilatum* did not exhibit any FISH signals (Fig. 2b–e).

The most reasonable explanation for the positive FISH signals with the B probe in the 5S rDNA region of A chromosomes, would be the presence of one or more repetitive DNA sequences associated with the 5S rDNA. As mentioned above, there is evidence of the involvement of 5S rDNA in the origin of satDNA in Bs of *P. lagopus* (Kumke et al. 2016), but as we still isolate no the satDNA fraction in *C. strigilatum*, aspects on the intern organization of this B is to be answered in future research. Besides that, 5S rDNA genes can be linked with different repeated sequences, in either coding or non-coding regions, as reviewed by Roa and Guerra (2015). Our results showed that although the conserved 5S rDNA fragment was amplified by PCR using a B chromosome as template, this DNA fraction could be in small copy numbers in the Bs. Besides, since the NTS region was not amplified by PCR using the isolated B as template, it is possible that 5S rDNA has been degraded in B, preventing the visualization of FISH signals. According to Houben et al. (1999), ribosomal DNAs are very dynamic sequences and cistron number can vary between B and A chromosomes.

**Table 1.** Results of Blast*n* alignment using 5S-Plant and UP primers PCR products against B chromosomes and genome of *Cestrum strigilatum*

Fragment	5S region	Subject	Accession	Cover	Ident.	*E-value*
5S-plant×Genome	NTS	*Cestrum aurantiacum* × *C. parqui*	AY135508.1	69%	76%	1e-60
5S-plant×Genome	NTS	*Cestrum psittacinum*	AF495752.1	42%	83%	2e-51
UP×Genome	gene coding	*Lilium tsingtauense*	KM117262.1	95%	96%	5e-44
UP×Genome	gene coding	*Vigna angularis*	AP017185.1	95%	96%	5e-44
UP×5S rDNA	gene coding	*Arabidopsis thaliana*	AY130622.1	100%	83%	2e-33
UP×B-chrom	gene coding	*Vigna angularis*	AP016873.1	92%	82%	6e-12
UP×B-chrom	gene coding	*Lilium tsingtauense*	KM117262.1	51%	92%	1e-07

### Detecting repetitive DNA families on A chromosomes

**Figure 4.**
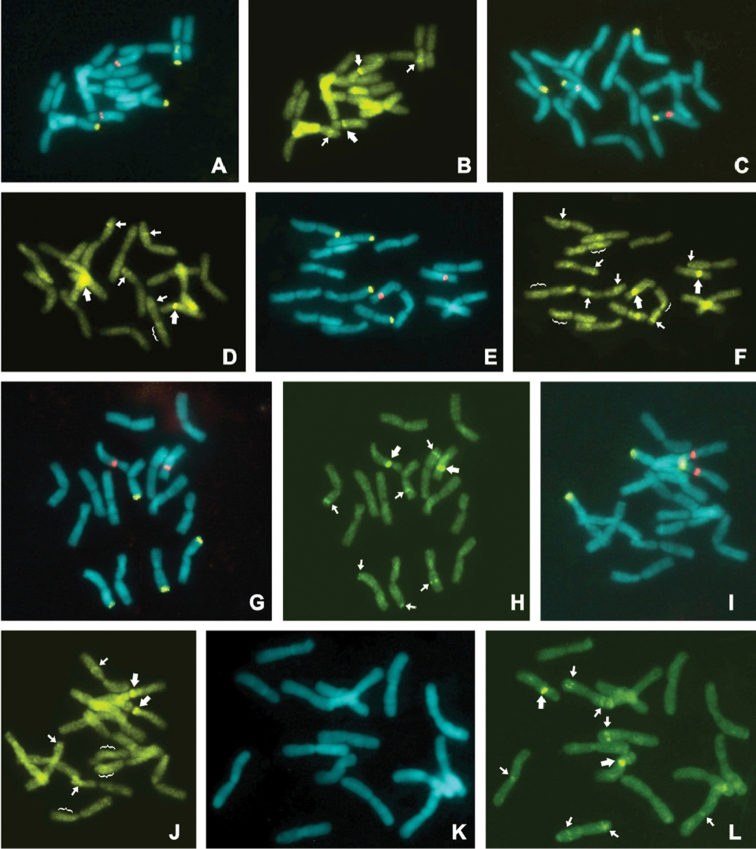
FISH showing the location of the 35S and 5S rDNA sites, and the diversity in the distribution of B probe signals in different species of *Cestrum*. All species showed four terminal 35S rDNA signals (green) and two proximal 5S rDNA signals (red). Note that in all cases, the 35S probe signals did not colocalize with B probe signals, but the 5S rDNA signals did with strong signals with B probe (large arrows). **A–B** *C. sendtnerianum*: the B probe also showed intercalary signals in one chromosome pair, without evidence of scattered hybridization signals. For other species **C–D** *C. corymbosum* **E–F** *C. laevigatum*, (Fig. 2D) **G–H** *C. schlechtendalii* **I–J** *C. bracteatum* (Fig. 2H) and **K–L** *C. mariquitense*, there was a variability in the number of intercalary hybridization signals (small arrows), besides small subterminal and terminal signals. Although all species showed scattered hybridization signals, it was more evident in chromosomes of *C. laevigatum* and *C. bracteatum* (brackets). The **K** image is only a conventional DAPI staining, without FISH with rDNA probes. Bar = 10 µm.

The B chromosome probe of *C. strigilatum* was also used to find complementarities in A chromosomes of six other *Cestrum* species (*C. bracteatum*, *C. corymbosum*, *C. laevigatum*, *C. mariquitense*, *C. sendtnerianum*, and *C. schlechtendalii*). FISH signals were detected in terminal, interstitial and proximal chromosome regions of these six species, but they varied in intensity, size and positioning between them. Intercalary signals were observed in one chromosome pair of *C. sendtnerianum* (Fig. 4b), two pairs of *C. corymbosum* (Fig. 4d), *C. schlechtendalii* (Fig. 4h) and *C. mariquitense* (Fig. 4l) and in three pairs of *C. laevigatum* (Fig. 4f) and *C. bracteatum* (Fig. 4j). Terminal hybridization signals were observed in one pair of *C. laevigatum* (Fig. 4f) and two pairs of *C. schlechtendalii* (Fig. 4h), and proximal signals were observed in one pair of *C. mariquitense* (Fig. 4l). All these FISH signals with the B probe support the idea that repetitive sequences present in different parts of A chromosomes may have been potentially adept to contributing for the formation and evolution of Bs. The example of Bs of *Brachycome dichromosomatica* (Asteraceae) is very illustrative, because the mini B chromosome contains a set of tandemly repetitive sequences are also found in A chromosomes, indicating that B can be involved in more than one event from the A complement (Houben et al. 2001, Jones and Houben 2003). Furthermore, the positive hybridization signals with this probe showed that although *Cestrum* species vary greatly in the distribution of repetitive DNA in karyotypes (Paula et al. 2015), some of these repetitive families are still conserved in the genus. In another example, rye B chromosomes share similar repeats with A chromosomes, differing in abundance retrotransposons (Klemme et al. 2013).

B chromosomes are generally under little or no selection pressure, and due to this, mobile elements and other repetitive DNA lineages may insert, spread, and amplify independently in these chromosomes (Houben et al. 2014). Possibly the dispensable nature of the Bs allowed the stronger chromosome diversification, such those reported for Bs of *C. parqui × C. aurantiacum*, *C. intermedium*, *C. strigilatum*, *C. parqui*, *C. euanthes* and *C. nocturnum* (Sýkorová et al. 2003, Fregonezi et al. 2004, Urdampilleta et al. 2015) for the presence and location of SSR, heterochromatin, *Gypsy* elements and rDNA between them. This feature is in accordance with the idea of specie-specific evolutionary pathways that were mentioned for the Bs (Houben et al. 2014). However, regardless of this diversification and the independent evolutionary history within each genome, this set of information could suggest that Bs have a recent origin in *Cestrum*, as they appear to retain similar sizes, and also because they share repetitive sequences with complement A chromosomes between different species, although we still do not know exactly the nature of this repetitive fraction. New investments in sequencing of isolated Bs should be next steps for provided us a better understand of B chromosomes organization, as well as to reveal details of the repetitive sequences roles in the arising of Bs.

## Supplementary materials

Supplementary material 1 Figure S1 Link: https://doi.org/10.3897/CompCytogen.v11i3.13418.suppl1 Data type: JPEG file Explanation note: **A**) Alignment of partial sequences of 5S rDNA of PCR product using the UP primers with *C. strigilatum* genome as template and PCR product using *C. strigilatum* B chromosome as template. Note the two internal deletions in the B chromosome amplicon, and other indels indicating a difference of 26%. **B**) Alignment of partial sequences of 5S rDNA of *C. aurantiacum* × *C. parqui*, AY135508.1 against PCR products using the 5S-Plant primers with *C. strigilatum* genome as template. Note an identity of 76% among them. This dataset is made available under the Open Database License (http://opendatacommons.org/licenses/odbl/1.0/). The Open Database License (ODbL) is a license agreement intended to allow users to freely share, modify, and use this Dataset while maintaining this same freedom for others, provided that the original source and author(s) are credited.André Luís Laforga Vanzela, Adriano Alves de Paula, Carolina Cristina Quintas, Thiago Fernandes, Joana Neres da Cruz Baldissera1, Thaíssa Boldieri de Souza
