# Supplementary figures and images for: Cestrum strigilatum (Ruiz & Pavón, 1799) B chromosome shares repetitive DNA sequences with A chromosomes of different Cestrum (Linnaeus, 1753) species
Source: Comp Cytogenet. 2017 Aug 3;11(3):511–24. doi: 10.3897/CompCytogen.v11i3.13418 (PMC5672077; doi:10.3897/CompCytogen.v11i3.13418)

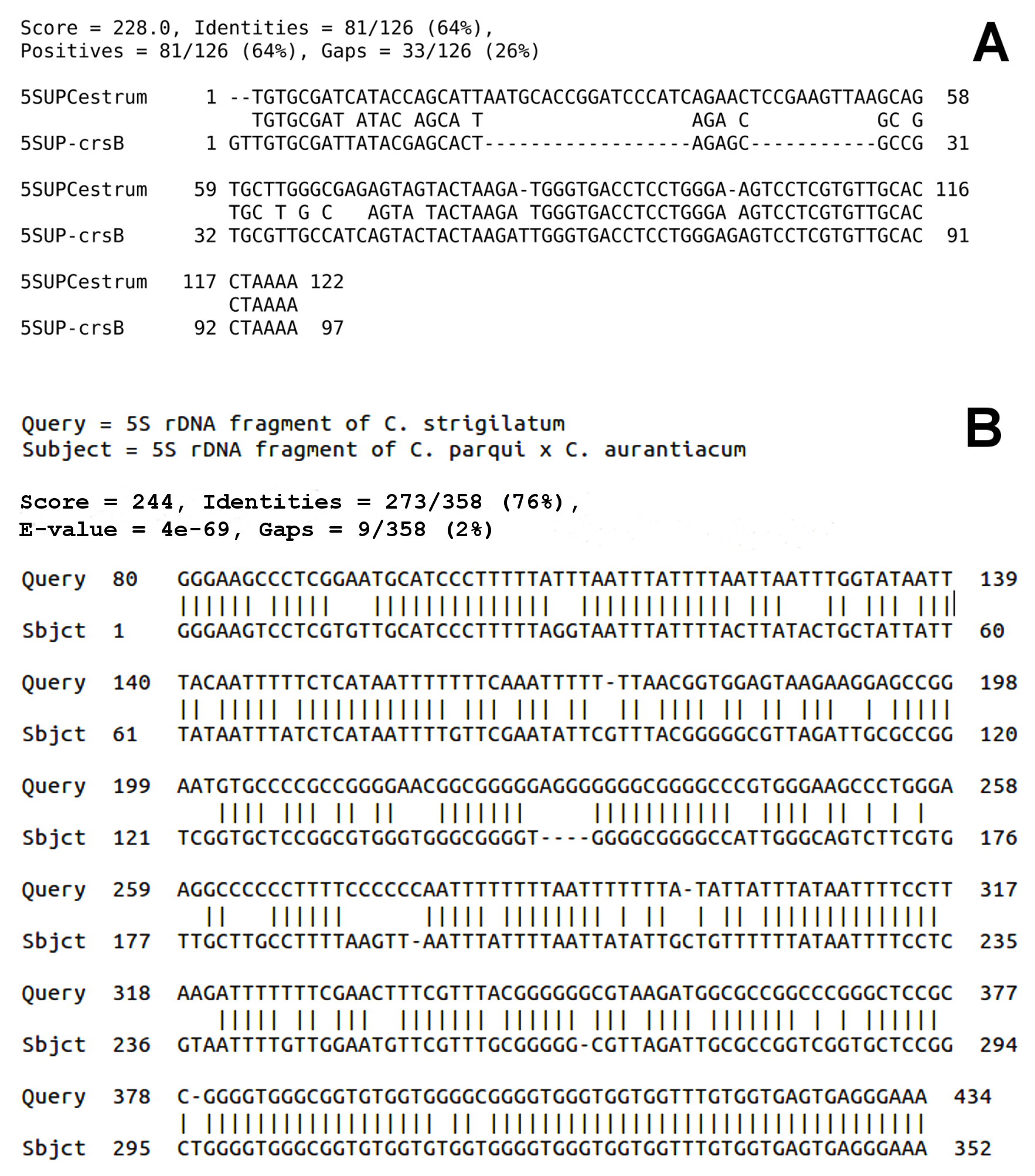

Supplement: Supplementary material 1 — Figure S1 [file comparative_cytogenetics-11-511-s001.jpg]
